# Safety and Efficacy of Eyevisc™ 2.4% Ophthalmic Viscosurgical Device in Cataract Surgery

**DOI:** 10.7759/cureus.112377

**Published:** 2026-07-09

**Authors:** Shreyas Ramamurthy, Dandapani Ramamurthy, Bhargav Joshi

**Affiliations:** 1 Ophthalmology, LASIK and Cataract Centre, The Eye Foundation Eye Hospital, Coimbatore, IND; 2 Vitreoretinal Surgery, LASIK and Cataract Centre, The Eye Foundation Eye Hospital, Coimbatore, IND; 3 Clinical Affairs, Biotech Vision Care Pvt. Ltd, Ahmedabad, IND

**Keywords:** anterior chamber surgery, cataract patients, cataract surgery, dispersive ophthalmic viscosurgical device (ovd), endothelial cell density, hydroxypropyl methylcellulose, intraocular pressure, ophthalmic viscosurgical device (ovd), visual acuity

## Abstract

Introduction

Hydroxypropyl methylcellulose (HPMC) is a dispersive ophthalmic viscosurgical device (OVD) that provides effective endothelial protection and anterior chamber stability during cataract surgery. However, clinical evidence for individual HPMC formulations remains limited. This study assessed the safety and efficacy of HPMC-based Eyevisc™ 2.4% (Biotech Vision Care Pvt. Ltd., Gujarat, India) in patients undergoing cataract surgery.

Methods

This was a prospective, single-arm, single-center, open-label clinical study that included patients undergoing cataract surgery, with the primary safety endpoint defined as subjects experiencing at least one postoperative intraocular pressure (IOP) measurement ≥30 mmHg. Other measured factors were endothelial cell density (ECD), visual acuity, corneal thickness, corneal clarity, and intraocular inflammation. Postoperative assessments were conducted at 8 hours (±2 hours), 24 hours (±4 hours), 7 days (±2 days), 30 days (±7 days), and 90 days (±14 days).

Results

The study included 131 (62 male and 69 female patients) with a mean age of 62 ± 8.16 years. No patient experienced an IOP spike of ≥30 mmHg at any follow-up with a mean of 20.26 ± 1.32 mmHg at 90 days. ECD was 2531.28 ± 237.31 cells/mm^2^ preoperatively, which remained preserved at the end of the study. Uncorrected and corrected distance visual acuity improved from 0.49 ± 0.27 logMAR and 0.25 ± 0.20 logMAR to 0.02 ± 0.04 logMAR and 0.00 ± 0.02 logMAR, respectively, postoperatively at 90 days (p<0.001, for both). Both corneal clarity and resolution of intraocular inflammation were achieved by 24 hours post-operatively. The surgeon-specific rating was excellent for the "Ease of use" in all cases, and also "Full chamber maintained" was observed in all cases (100%). The study reported no adverse event.

Conclusion

The study observed no case of the IOP spike, and the ECD was well preserved post-operatively, strongly suggesting the safety and effectiveness of Eyevisc™ 2.4% in patients undergoing cataract surgery.

## Introduction

Cataract surgery is one of the most frequently performed surgical procedures worldwide and is highly effective in restoring visual functions. Modern phacoemulsification with intraocular lens (IOL) implantation has significantly enhanced surgical precision and improved refractive outcomes of cataract surgery. However, ultrasonic energy, irrigation turbulence, and intraocular manipulation may induce corneal endothelial stress and transient postoperative inflammation, potentially affecting early visual recovery and the surgical safety of phacoemulsification [1].

The cornea is a transparent, avascular, and richly innervated tissue that plays a critical role in transmitting light to the retina and maintaining corneal homeostasis. The innermost layer, the corneal endothelium, comprises a single layer of polygonal cells responsible for maintaining corneal transparency and stromal hydration through active fluid regulation. The corneal endothelial cell density (ECD) is highest at birth, averaging approximately 3,000 cells/mm², and gradually declines to approximately 2,500 cells/mm² in adulthood. Given the limited regenerative capacity of endothelial cells, preserving cell density is essential. A critical ECD threshold of approximately 400-500 cells/mm² is required to sustain endothelial pump function. When the ECD falls below this level, corneal deturgescence cannot be maintained, leading to stromal edema, loss of transparency, and a consequent reduction in visual acuity [2,3]. Thus, preservation of the corneal endothelium is critical during cataract surgery.

Ophthalmic viscosurgical devices (OVDs) play a critical role in contemporary cataract surgery by maintaining anterior chamber stability, facilitating capsulorhexis and IOL implantation, and protecting intraocular structures, particularly the corneal endothelium [4-6]. Dispersive agents such as Eyevisc^TM^ are characterized by their superior endothelial coating properties, enabling effective intraoperative tissue protection and chamber stability [7]. Appropriate surgical techniques and thorough removal of the lens at the conclusion of the procedure are essential to ensure optimal postoperative intraocular pressure (IOP) control. Postoperative IOP monitoring remains a standard safety consideration following cataract surgery, irrespective of the OVD class. [8]

Eyevisc™ (Biotech Vision Care Pvt. Ltd., Gujarat, India) is a sterile, non-pyrogenic, viscoelastic preparation of a non-inflammatory, highly purified grade of hydroxypropyl methylcellulose (HPMC) 2.4% w/v dissolved in a physiological buffer at pH 6.8-7.6 for intraocular application during anterior segment surgeries. It is a highly retentive and dispersive viscoelastic agent with its unique ability to offer optimal space maintenance and excellent tissue protection throughout the procedure [9]. The present study assesses the safety and efficacy of Eyevisc™ 2.4% in patients undergoing cataract surgery, fulfilling the requirement for targeted data regarding this specific ocular viscoelastic device.

## Materials and methods

Study design and patients

This prospective, single-arm, single-center clinical study was conducted between September 2023 and May 2024 at The Eye Foundation, Coimbatore, Tamil Nadu, India. The ethical committee of Eye Foundation, Coimbatore, approved the study (protocol no. BVCPL-HPMC-2023-01), and all participants provided written informed consent prior to its initiation. The study was conducted in accordance with the principles of the Declaration of Helsinki and ISO 14155 and was registered with the Clinical Trials Registry-India (CTRI/2023/11/060182).

The study enrolled Grade I to III cataract patients as per the simplified cataract grading system [10], aged ≥45 years, for whom phacoemulsification extraction and posterior chamber IOL implantation were planned in at least one eye. Patients with pupil dilation greater than 7.0 mm and clear intraocular media apart from cataracts were included in the study. Additionally, the study included patients who signed informed consent and were willing to attend all regular follow-up examinations. Patients with a history of previous steroid-induced IOP, intraocular or corneal surgery, or ocular trauma were excluded. Further patients with pigment dispersion syndrome, pseudoexfoliation, corneal abnormalities, and chronic or recurrent inflammatory eye disease were excluded from the study.

Endpoint

The primary safety endpoint was defined as the proportion of subjects who experienced at least one IOP measurement ≥30 mmHg in the study eye at any postoperative follow-up visits, and the primary effectiveness endpoint was the percentage change in ECD from baseline to the fifth postoperative visit (3 months ± 14 days) in the study eye.

Preoperative and postoperative examination

Participants underwent evaluations at the following time points: preoperative (screening), intraoperative (surgery visit), and postoperatively at 8 hours (±2 hours), 24 hours (±4 hours), 7 hours (±2 days), 30 days (±7 days), and 90 days (±14 days). IOP was measured at all visits using Goldmann applanation tonometry. Corneal ECD (cells/mm²), coefficient of variation (CV) in cell size, mean cell area, and percentage of hexagonal cells (hexagonality) were assessed preoperatively and at 90 days postoperatively using Tomey Specular Microscope (EM-2000). CV was used to quantify variability in endothelial cell size. Central corneal thickness was measured preoperatively and at seven days, 30 days, and 90 days postoperatively using the SIRIUS topographer (CSO SRL, Firenze, Italy). Intraocular inflammation was evaluated at eight hours, 24 hours, seven days, 30 days, and 90 days using slit-lamp biomicroscopy, and grading was performed according to the Standardization of Uveitis Nomenclature (SUN) [11] based on aqueous cells and flare. Distance visual acuity was assessed preoperatively and at 30 days and 90 days using standardized Early Treatment Diabetic Retinopathy Study (ETDRS) charts at 4 meters. Slit-lamp biomicroscopy and corneal haze grading were used to check the clarity of the cornea at all postoperative visits [12]. Intraoperative parameters, including total OVD stay time (min) as well as surgeon-specific ratings for ease of use (Excellent/Very good/Good/Need to improve) and chamber maintenance (Full chamber/Working space/Shallow/Flat), were also recorded. Adverse events were recorded intraoperatively, at each postoperative visit, and whenever they occurred during the study period.

Surgical procedure

All procedures were performed by a single, experienced surgeon to ensure standardization. A 2.2-mm clear corneal incision with a side port was made along the steepest meridian of the cornea while the patient was under topical anesthesia (0.5% proparacaine). Then Eyevisc™ 2.4% was injected into the anterior chamber to maintain space and protect intraocular tissues during continuous curvilinear capsulorhexis and phacoemulsification using the crater-and-chop technique. Following cortical aspiration, the capsular bag was re-expanded with viscoelastic material prior to the implantation of a single-piece acrylic foldable intraocular lens. The OVD was removed at the end of the surgery to minimize the postoperative IOP elevation. Moxifloxacin eye drops (Moxflu®) were used as perioperative antibiotic prophylaxis. No periocular corticosteroids, subconjunctival injections, or additional intraocular medications (e.g., adrenaline, acetylcholine, or carbachol) were administered. The characteristics of Eyevisc™ 2.4% are presented in Table 1.

**Table 1:** Eyevisc™ 2.4% device description Eyevisc™ 2.4% (Biotech Visioncare Pvt. Ltd., Gujarat, India).

S No.	Parameters	Description
1	Composition	Hydroxypropyl Methylcellulose USP: 2.4% w/v Sterile Isotonic base: Q.S.
2	Viscosity	Between 7500 mPas to 12500 mPas at 25°C ± 2°C
3	Osmolality	270 – 400 mOsm/kg
4	Molecular weight	Greater than 80,000 Daltons
5	pH	6.8 to 7.6
6	Storage condition	Store between 2°C to 35°C

Statistical analysis

The sample size was estimated using PASS 2021 software (NCSS, LLC., Utah, USA) based on a reference proportion of 7.2%, an assumed proportion of 1.0%, 90% power, and a two-sided α of 0.05. Allowing for a 20% dropout rate, 131 subjects were planned for enrollment to ensure at least 105 evaluable subjects for the effectiveness analysis. Continuous variables are summarized as mean ± standard deviation (SD) or median (range), and categorical variables as frequencies and percentages. Between-group comparisons were performed using analysis of covariance (ANCOVA) or the Mann-Whitney U test, as appropriate, based on data distribution. Within-group comparisons were assessed using paired t-tests or Wilcoxon signed-rank tests as appropriate. Statistical significance was set at p<0.05. Statistical analyses were performed using SAS version 9.4 or later (SAS Inc., Cary, NC, US).

## Results

**Figure 1:**
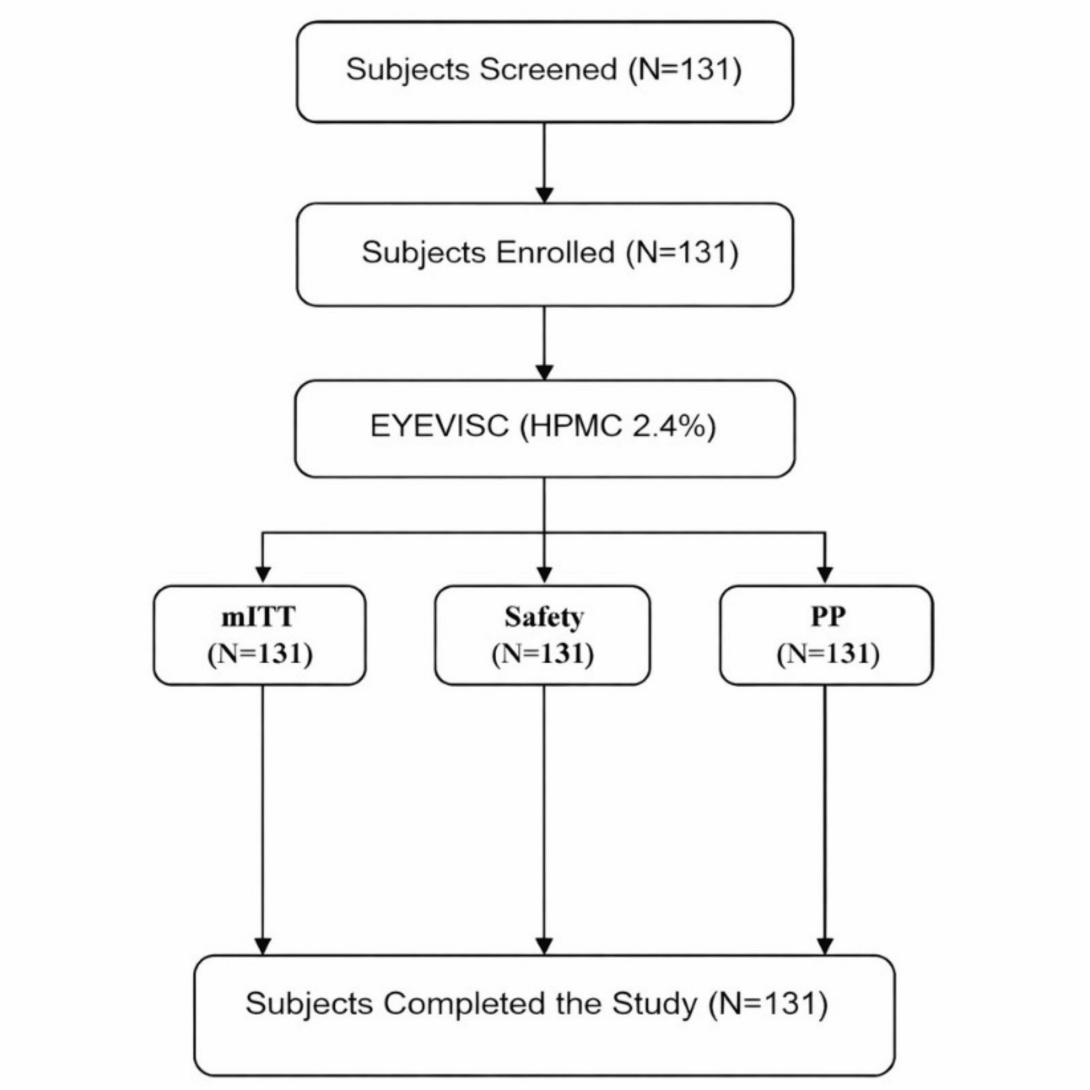
Subject disposition flow diagram showing patient screening, enrollment, analysis populations, and study completion in the clinical evaluation of Eyevisc™ 2.4% during cataract surgery mITT: modified intent-to-treat population; PP: per-protocol population.

A total of 131 subjects were screened and enrolled in the study, of which 62 (47.33%) were men and 69 (52.67%) were women, aged between 45 and 80 years, with a mean (SD) age of 62 (8.16) years. All patients completed the study, and no discontinuations were reported. (Figure 1).

The baseline characteristics of the study population are listed in Table 2.

**Table 2:** Baseline demographic and preoperative ocular characteristics of patients undergoing cataract surgery using Eyevisc™ 2.4% Data are presented as mean ± standard deviation (SD) or number (%). IOP: intraocular pressure; UDVA: uncorrected distance visual acuity; CDVA: corrected distance visual acuity.

S No.	Characteristics	Eyevisc™ 2.4%
1	Patient (N)	131
2	Age (in years); Mean (SD)	62(8.16)
3	Sex, n (%)	
	Male	62 (47.33)
	Female	69 (52.67)
4	IOP (mmHg) Mean ± SD	13.54 ± 2.07
5	Endothelial Cell Density (cells/mm²)	2531.28 ± 237.31
6	Corneal Thickness (µm)	536.35 ± 37.39
7	UDVA (logMAR)	0.49 ± 0.27
8	CDVA (logMAR)	0.25 ± 0.20
9	Coefficient of cell size variation (%)	42.24 ±12.30
10	Cell Area (µm²)	398.81 ± 37.57
11	Cell Hexagonality (%)	44.38 ± 6.07

Intraocular pressure (IOP)

**Figure 2:**
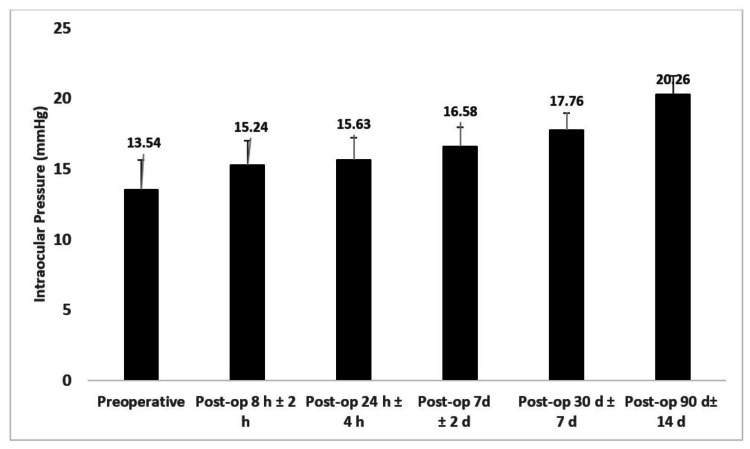
Mean intraocular pressure (IOP) measured preoperatively and at postoperative follow-up visits up to day 90 (±14 days) following cataract surgery using Eyevisc™ 2.4% Data are presented as mean ± standard deviation (SD).

No cases of IOP elevation ≥30 mmHg were observed during the study. The mean preoperative IOP was 13.54 ± 2.07 mmHg, with a modest increase in the early postoperative period to 15.24 ± 1.74 mmHg at eight hours and 15.63 ± 1.56 mmHg at 24 hours postoperatively. A gradual rise in mean IOP was noted over the follow-up period, reaching 16.58 ± 1.38 mmHg on day seven, 17.76 ± 1.18 mmHg on day 30, and 20.26 ± 1.32 mmHg at 90 days. Despite these statistically significant changes compared with the baseline (p<0.0001 at all postoperative visits), all recorded IOP values remained within clinically acceptable limits (Figure 2).

Endothelial cell density (ECD)

The preoperative ECD was 2531.28 ± 237.31 cells/mm², which increased to 2603.15 ± 187.57 cells/mm² at the end of the study (p<0.0001). As corneal endothelial cells have negligible regenerative capacity, this apparent increase is unlikely to represent a true biological change. It is more likely attributable to measurement variability associated with specular microscopy, influenced by differences in corneal optical quality between pre- and postoperative examinations. The observed increase (~2.8%) falls within the reported test-retest variability of specular microscopy and was therefore not considered clinically significant [13].

Endothelial cell morphology

**Figure 3:**
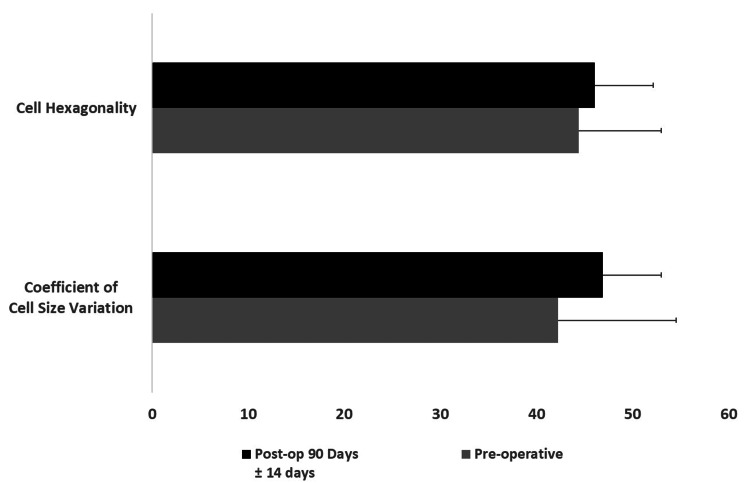
Comparison of endothelial morphological parameters Compares cell hexagonality (%) and coefficient of cell size variation between the preoperative visit and postoperative day 90 (±14 days) following cataract surgery using Eyevisc™ 2.4%. Data are presented as mean ± standard deviation.

Preoperatively, coefficients of cell size variation and cell hexagonality were 42.24% ± 12.30 and 44.38% ± 6.07, which increased to 46.91% ± 8.63 and 46.06% ± 6.13, respectively. The change was statistically significant only in cell hexagonality (p=0.003). The cell area followed the same trend; at preoperative, the mean value was 398.81 ± 37.57 µm², which increased to 458.04 ± 38.32 µm² (p<0.001). All the cell morphological parameters remain in the clinically normal range (Figure 3).

Visual acuity

Visual acuity showed a consistent improvement during the follow-up period. Preoperatively, the mean uncorrected distance visual acuity (UDVA) and corrected distance visual acuity (CDVA) were 0.49 ±0.27 logMAR and 0.25 ±0.20 logMAR, respectively, improving to 0.29 ±0.21 logMAR and 0.13 ±0.13 logMAR at seven days postoperatively. The visual acuity further improved to 0.15 ± 0.16 logMAR and 0.06 ± 0.09 logMAR at 30 days, respectively. At 90 days, UDVA and CDVA reached 0.02 ± 0.04 and 0.00 ± 0.02 logMAR, respectively. The intra-group p-values showed statistical significance at each visit (p<0.001) for both the visual acuities. 

Corneal thickness

The corneal thickness increased during the follow-up period. The preoperative mean ± SD 536.35 ± 37.39 µm increased to 544.47 ± 32.14 µm, 553.15 ± 31.96 µm, and 567.34 ± 28.88 µm at seven days, 30 days, and 90 days, respectively (p<0.001, all visits). Although each visit showed statistically significant changes, the corneal thickness remained within the clinically acceptable range postoperatively.

Corneal clarity

At the preoperative visit, most of the subjects had Grade 0, indicating no significant issues. At the postoperative visits, the majority of subjects had Grade 0 across all time points: 99.24% at eight hours and 100% at the remaining follow-ups. Only 0.76% of the subjects presented with Grade 1 at eight hours ± two hours postoperatively, but no subjects exhibited Grade 1 or higher at 24 hours postoperatively.

Intraocular inflammation

By 24 hours after the operation, the majority of subjects demonstrated complete resolution of aqueous cells and flare, with 100% of subjects showing no aqueous cells or flare, and no higher-grade cases were observed.

Intraoperative parameter

During surgery, the OVD remained in the anterior chamber for a mean duration of 4.45 ± 0.92 minutes. The surgeon rated the OVD as excellent for ease of use in all cases. Also, chamber maintenance was full chamber maintenance in all cases (100%).

Adverse event

There were no adverse events observed during the study.

## Discussion

The present study was a prospective, single-arm clinical study that affirmed the safety and efficacy of Eyevisc™ 2.4%, an HPMC-based OVD, in patients undergoing cataract surgery. No patient experienced an IOP ≥ 30 mmHg at any of the postoperative visits. A statistically significant increase in mean IOP was observed from 13.54 ± 2.07 mmHg at baseline to 20.26 ± 1.32 mmHg at 90 days (p<0.0001). However, the IOP values remained within physiological limits, suggesting that the OVD was effectively removed and did not compromise trabecular outflow.

The IOP profile observed in the present study compares favorably with reports of other OVDs in the literature. For instance, Ocucoat® (2% HPMC) has been associated with transient IOP spikes within the first eight postoperative hours, while another 2% HPMC formulation demonstrated elevated IOP in approximately 30% of treated eyes [5,14]. Furthermore, in comparison with sodium hyaluronate (NaHA)-based OVDs, a meta-analysis reported fewer postoperative IOP spikes with HPMC in eyes undergoing phacoemulsification. Another study found no statistically significant difference between HPMC and NaHa-CS in IOP at any point post-surgery [15].

Corneal ECD changed from 2531.28 ± 237.31 cells/mm² at baseline to 2603.15 ± 187.57 cells/mm² at 90 days, with the difference reaching statistical significance (71.87 ± 122.39 cells/mm²; p<0.0001). Although this numerical increase is not physiologically expected, it is likely due to measurement variability or imaging artifacts rather than a true biological gain. Importantly, the data indicate that no clinically significant ECD loss occurred, suggesting that Eyevisc™ 2.4% provided effective endothelial protection during surgery, as reported in literature where 2% HPMC showed higher ECD loss than 2.4% post-cataract surgery [16]. However, the HPMC group reported a significantly higher post-operative mean corneal cell count at one week, one month, and three months compared to the NaHA OVD group [17]. Furthermore, a meta-analysis using a random-effects model revealed a significantly lower percent decrease in ECD for chondroitin sulfate-hyaluronic acid (CS-HA) OVDs compared to both HA-only (MD: −4.10%; 95% CI: −5.81 to −2.40; p<0.0001; nine studies) and HPMC (MD: −6.47%; 95% CI: −10.41 to −2.52; p = 0.001; two studies) products [18].

Corneal thickness increased progressively from a preoperative value of 536.35 ± 37.39 µm to 567.34 ± 28.88 µm at 90 days postoperatively (p<0.001), while remaining within a clinically acceptable range. These findings are consistent with previous studies evaluating OVDs. In a comparative study of DiscoVisc and 2% HPMC, preoperative corneal thickness was 535.3 ± 33.9 µm and 538.2 ± 35.9 µm, respectively, increasing to 543.2 ± 36.8 µm and 543.8 ± 39.5 µm at 90 days postoperatively [19]. Similarly, another study comparing dispersive and cohesive OVDs reported an increase in corneal thickness from 549.10 ± 19.08 µm and 560.45 ± 16.72 µm preoperatively to 586.75 ± 20.43 µm and 595.00 ± 19.73 µm, respectively, two months postoperatively [14].

Complete resolution of intraocular inflammation (Grade 0 cells and flare) and corneal clarity were observed in all subjects by 24 hours postoperatively, indicating their safety during the early postoperative period. The findings are better than those of cohesive-dispersive OVDs such as Duovisc (Alcon Laboratories, Fort Worth, US) and Viscopack (CIMA Technology Inc. , Pittsburgh, US), in which intraocular inflammation was observed until 24 hours and resolved within seven days [20]. The postoperative period improved other functional outcomes, such as visual acuity. Preoperatively, mean UDVA and CDVA were 0.49 ± 0.27 logMAR and 0.25 ± 0.20 logMAR, respectively, improving to 0.02 ± 0.04 logMAR and 0.0 ± 0.02 logMAR at 90 days (p<0.001 for both visual acuities). The result was consistent with the findings of various OVDs reported in the literature [6]. Further, no serious or device-related adverse events were reported. The observed minor postoperative events resolved with standard postoperative care and did not require additional medical or surgical intervention, supporting the favorable safety profile of Eyevisc™ 2.4%.

The study has limitations, including a single-arm design without a comparator and being conducted at a single center, which may limit the generalizability of the population. Despite the study's limitations, these findings collectively provide the first dedicated clinical evidence that Eyevisc™ 2.4% demonstrates a highly favorable safety and efficacy profile against current standards for OVD performance during cataract surgery, particularly through robust endothelial protection, minimal postoperative inflammation, and excellent visual outcomes.

## Conclusions

This study provides crucial clinical evidence demonstrating the safety of Eyevisc^TM^ 2.4%, with no observed cases of elevated IOP or other adverse events, thereby establishing a favorable safety profile for cataract surgery. ECD was well-preserved post-surgery, and other endothelial cellular morphological parameters remained within clinically acceptable ranges. The cornea remained completely clear, and no intraocular inflammation was observed after 24 hours. Visual acuity (UDVA and CDVA) significantly improved at each follow-up visit, and the surgeon provided an excellent rating for ease of use and chamber maintenance, which collectively summarize the safety and efficacy of Eyevisc™ 2.4% in cataract surgery.
